# Inflammatory, Oxidative Stress and Small Cellular Particle Response in HUVEC Induced by Debris from Endoprosthesis Processing

**DOI:** 10.3390/ma16093287

**Published:** 2023-04-22

**Authors:** Zala Jan, Matej Hočevar, Veno Kononenko, Sara Michelini, Neža Repar, Maja Caf, Boštjan Kocjančič, Drago Dolinar, Slavko Kralj, Darko Makovec, Aleš Iglič, Damjana Drobne, Monika Jenko, Veronika Kralj-Iglič

**Affiliations:** 1 University of Ljubljana, Faculty of Health Sciences, Laboratory of Clinical Biophysics, SI-1000 Ljubljana, Slovenia; 2 Institute of Metals and Technology, SI-1000 Ljubljana, Slovenia; 3 University of Ljubljana, Biotechnical Faculty, Nanobiology Group, SI-1000 Ljubljana, Slovenia; 4 Department for Materials Synthesis, Jožef Stefan Institute, SI-1000 Ljubljana, Slovenia; 5 University of Ljubljana, Faculty of Pharmacy, SI-1000 Ljubljana, Slovenia; 6 University of Ljubljana, Faculty of Medicine, Chair of Orthopaedics, SI-1000 Ljubljana, Slovenia; 7 MD-RI Institute for Materials Research in Medicine, SI-1000 Ljubljana, Slovenia; 8 Department of Orthopaedic Surgery, University Medical Centre Ljubljana, SI-1000 Ljubljana, Slovenia; 9 University of Ljubljana, Faculty of Electrical Engineering, Laboratory of Physics, SI-1000 Ljubljana, Slovenia; 10 University of Ljubljana, Faculty of Medicine, Laboratory of Clinical Biophysics, SI-1000 Ljubljana, Slovenia

**Keywords:** endoprosthesis failure, corundum, cytokines, reactive oxygen species, lipid droplets

## Abstract

We studied inflammatory and oxidative stress-related parameters and cytotoxic response of human umbilical vein endothelial cells (HUVEC) to a 24 h treatment with milled particles simulating debris involved in sandblasting of orthopedic implants (OI). We used different abrasives (corundum—(Al_2_O_3_), used corundum retrieved from removed OI (u. Al_2_O_3_), and zirconia/silica composite (ZrO_2_/SiO_2_)). Morphological changes were observed by scanning electron microscopy (SEM). Concentration of Interleukins IL-6 and IL-1β and Tumor Necrosis Factor α (TNF)-α was assessed by enzyme-linked immunosorbent assay (ELISA). Activity of Cholinesterase (ChE) and Glutathione S-transferase (GST) was measured by spectrophotometry. Reactive oxygen species (ROS), lipid droplets (LD) and apoptosis were measured by flow cytometry (FCM). Detachment of the cells from glass and budding of the cell membrane did not differ in the treated and untreated control cells. Increased concentration of IL-1β and of IL-6 was found after treatment with all tested particle types, indicating inflammatory response of the treated cells. Increased ChE activity was found after treatment with u. Al_2_O_3_ and ZrO_2_/SiO_2_. Increased GST activity was found after treatment with ZrO_2_/SiO_2_. Increased LD quantity but not ROS quantity was found after treatment with u. Al_2_O_3_. No cytotoxicity was detected after treatment with u. Al_2_O_3_. The tested materials in concentrations added to in vitro cell lines were found non-toxic but bioactive and therefore prone to induce a response of the human body to OI.

## 1. Introduction

With the increased use of orthopedic implants (OI) due to increased life expectancy, biocompatibility and relevance of materials are gaining interest. OI materials must be biologically acceptable to minimize adverse local tissue reactions and robust enough to support weight bearing during common activities of daily life [1]. Modern materials for joint replacement are well tolerated and accepted by the body if they are in bulk form, mechanically stable and sterile [1]. However, it was found that fibrotic tissue often surrounds surgical implants, which was connected to metallic wear particles released into the tissue that surrounds OI [2]. Tissue damage can trigger inflammation which can result in fibrosis through different pathways [3]. Excessive wear of OI that produces particle debris (and consequently, osteolysis) results in aseptic loosening of the OI. Pain and reduced mobility indicate a revision surgery [4,5] that represents an additional risk to patients, also regarding thromboembolic events, infection, dislocation and death [6]. The choice of the materials used and surface elaboration are therefore key to the longevity of the endoprosthesis. 

The main materials used for endoprostheses are metal, polyethylene and ceramic. Follow-up studies of populations of hips with an implanted prosthesis showed different survival rates and different problems with different materials and their combinations (reviewed in [7]). Ceramics showed promising results regarding wear and related loosening; a 6 year (mid-term) follow-up study including 310 hips with ceramic head and liner prostheses showed that 99.0% of the hips had not been associated with re-operation and there was no radiological evidence of osteolysis or loosening [8]. It was reported that alumina−alumina ceramic OI generated 400 times fewer wear particles than metal-polyethylene OI, which resulted in a lower rate of periprosthetic osteolysis in alumina−alumina OI [9]. Zirconia-toughened alumina ceramic was found to release 1 µg/year of wear debris into circulation and surrounding tissue, which is considered very low [10].

The quality of the prostheses could be improved by focusing on the microscopic properties of the interfaces (between the prosthesis parts and between the prosthesis and tissues). In ceramics, most of the debris was found to consist of particles sized between 0.1 and 10 μm, but larger particles sized up to 1 mm were also observed [11]. Besides toxic effects, also inflammatory and oxidative stress responses are of importance. It was summarized in the review on the response of cells that, upon biomaterial implantation, a sequence of events is initiated with an injury, followed by blood−material interactions, provisional matrix formation and acute innate inflammatory response acting on monocytes, fibroblasts, osteoblasts, osteoclasts and mesenchymal stem cells [12]. The activation of macrophages was suggested as the dominant mechanism in periprosthetic inflammation [13]. It begins with interaction of the particles on membrane receptors (such as CD14 and toll-like receptors) followed by the release of pro-inflammatory cytokines (e.g., tumor necrosis factor (TNF)-α, IL-1β, IL-6, prostaglandin E (PGE)-2), growth factors (macrophage colony stimulating factor 1—M-CSF), pro-osteoclastic factors (receptor activator of nuclear factor kappa B ligand—RANKL) and chemokines (e.g., IL-8, macrophage inflammatory protein—MIP-1α, monocyte chemoattractant protein—MCP-1). Moreover, phagocytosis of wear debris takes place [12]. The suggested underlying mechanisms are the up regulation of transcription factor NF κβ and the activation of inflammasome danger signaling. This leads to a decreased osteoblast function and increased osteoclast activity [14]. It was suggested that inefficient phagocytosis with excessive production of inflammatory mediators may lead to sustained inflammation and, eventually, fibrotic changes [3,15].

In a study involving murine macrophages (RAW264.7) exposed to corundum micro- and nanoparticles, aliquots of cell culture supernatants were tested for different cytokines, growth factors and nitric oxide [16]. Exposure to corundum particles led to a decrease in the number of vital macrophages, an increase in the number of giant cells, formation of micron-sized aggregates in the cell culture medium and production of giant cells [16].

Human articulate chondrocytes were shown to attach to composite Al_2_O_3_, SiO_2_, CaAl_2_Si_2_O_8_, Ca_3_ (PO_4_)_2_, Ca_2_Al_4_O_7_ and NaAlSiO_4_ surfaces [17]. However, elaboration of the surface turned out to be of importance. It was shown that corundum sandblasting of surfaces significantly increased surface wettability, MG63 cell attachment and proliferation and alkaline phosphatase activity in comparison with the control surface [18]. The corundum contamination was found on and under the surface of the new and retrieved dental implants [19]. The study of new and retrieved dental implants and restorative materials—commercially pure titanium (cpTi), the Ti_6_Al_4_V alloy and CoCrMo—by light microscopy, SEM and energy-dispersive spectroscopy, showed that the surfaces of the Ti and Ti_6_Al_4_V implants were affected by corundum blasting [20,21]. Moreover, contamination of the new and early removed femoral components made of the alloy Ti_6_Al_7_Nb with corundum wear particles was found also 5 to 20 μm below the surface [22], as the hard corundum wear particles were embedded into a softer matrix of Ti_6_Al_7_Nb alloy during the sandblasting process. The microstructural analysis of the cross-sections showed that the cracks around the built-in particulate matter range to the surface. Such cracks allow corrosion and represent sites for the attachment and colonization of bacteria, the formation of biofilm, periprosthetic infection and implant failure. Therefore, in addition to aseptic loosening of the implant, the corundum particles can cause periprosthetic infection [22]. As unavoidable generation of wear debris from any part of a prosthesis leads to prosthesis failure, histological analysis of the tissue obtained during implant revision surgery is considered important for wear-particle identification, and for the classification of biological reactions to wear particles [23].

It is therefore important to study the effect of debris on cells. In a preliminary study, we observed morphological changes in HUVEC to exposure to corundum particles [24]. With the aim of better understanding the mechanisms underlying the effects of the sandblasting debris contamination on osteointegration, we here address the effect of three types of particles: Al_2_O_3_—white alumina; u. Al_2_O_3_—white alumina previously used in the process of sandblasting of OI; and ZrO_2_/SiO_2_—zirconia/silica composite, on morphology, inflammatory response, oxidative stress response and cytotoxicity and of HUVEC. 

## 2. Materials and Methods

### 2.1. Cell Culture

HUVEC were a kind gift of Snežna Sodin Šemrov from the Immunology Laboratory, Department of Rheumatology, University Medical Centre Ljubljana, Ljubljana, Slovenia. They were purchased from Lonza, Basel, Switzerland, No. 480242. HUVEC were confirmed to be mycoplasma negative using the MycoAlert™ Kit (Lonza, Basel, Switzerland). Cells were cultured with an initial concentration of 3 × 10^4^ cells/cm^2^ and allowed to attach and grow for 24 h in Dulbecco’s modified Eagle’s medium (Sigma Aldrich, St. Louis, MO, USA), supplemented with 4 mM L-glutamine and 5% (*v*/*v*) fetal bovine serum (FBS) (Sigma Aldrich, St. Louis, MO, USA) at 37 °C in an incubator with a humidified atmosphere containing 5% CO_2_. For ROS assay positive controls, cells were treated with 5 mM H_2_O_2_ for 30 min. For LD assay positive controls, cells were treated with 75 μM oleic acid for 24 h. For apoptosis positive control, cells were treated with Staurosporine 10 µM for 24 h. Experiments with cells were made in a duplicate or triplicate.

### 2.2. Preparation of Particles

Three different types of particles: Al_2_O_3_, white alumina; u. Al_2_O_3_, white alumina previously used in the process of sandblasting of OI; and ZrO_2_/SiO_2_ were obtained from FerroECOBlast, Dolenjske Toplice, Slovenija. Original-sized particles were milled in smaller particles by shaking the samples with 2 cm diameter steel beads at 1500 Hz for 10 min in the shaker Milimix 20, Domel, Slovenia. Prior to being added to the cell culture media, the particles were sterilized using UV light.

### 2.3. Measurement of Zeta Potential

The suspensions of particles were monitored with electro-kinetic measurements of the ζ-potential [25] by using a Litesizer™ 500 (Anton Paar GmbH, Graz, Austria). The values of the ζ-potential were measured in particle suspension containing either fresh or conditioned cell medium at final particle concentration of 100 μg/mL. Before each individual measurement, the pH value of the suspension was determined.

### 2.4. Dynamic Light Scattering (DLS)

The hydrodynamic diameter of particles (D_h_) in fresh and conditioned cell media was determined by dynamic light scattering (DLS) [26] using a Litesizer™ 500 (Anton Paar GmbH, Graz, Austria). The D_h_ values were obtained from the diffusion coefficients (D) that were assessed from the correlation function of the scattered electric field (g_1_(t)) obtained from the correlation function of the scattered light intensity g_2_(t) by applying the Siegert relation. To convert D to D_h_, the Stokes−Einstein equation was used (D_h_ = kT/3πηD, where k is the Boltzmann constant, T is the absolute temperature and η is the viscosity of the medium in which the particles diffused). The viscosity of the medium was approximated to the viscosity value of water at 25 °C. The scattered light was measured at an angle θ = 90°.

### 2.5. Characterization of Abrasives

The abrasive particles for sand blasting were characterized by a combination of X-ray powder diffraction [27] (XRD, PANalytical X’Pert Pro MPD diffractometer), scanning electron microscopy (SEM, Thermo Fisher Quanta 650) operated at 5 kV [28] and transmission electron microscopy (TEM, JEOL, JEM 2010 F, Akishima, Tokyo, Japan) operated at 200 kV [28] coupled with energy-dispersive X-ray spectroscopy (EDXS) [29]. For the SEM investigations, the particles were deposited on a conductive carbon tape and sputtered with 6 nm layer of Au-Pd. For the TEM investigations, the particles were deposited by drying a drop of suspension on a copper-grid-supported perforated transparent carbon foil. 

### 2.6. Treatment of Cells with Ceramic Particles

For treatment, the cell culture medium was replaced with respective media containing different concentrations of different particles (i.e., 10, 50 and 100 μg/mL). Cells were further grown under the same conditions for 24 h.

### 2.7. Measurements of Inflammation Processes by IL-6, IL-1β, and TNF-α

IL-6, IL-1β and TNF-α were measured as described in [30]. The solution was composed of equal volumes of conditioned medium and Sample Diluent Buffer A from the enzyme-linked immunosorbent assay (ELISA) kit (Sigma Aldrich, St. Louis, MO, USA) catalog numbers: SI-RAB306 for IL-6, SI-RAB0273 for IL-1β and SI-RAB0476 for TNF-α. A 100 μL sample was assessed spectrophotometrically by measuring the absorbance at 450 nm with BioTek (Cytation 3, Bad Friedrichshall, Germany) instrument. The results were expressed in pg/mL of the sample.

### 2.8. Cholinesterase Activity Assay

Treated cells (cca 3 × 10^4^ cells/cm^2^) were evaluated for ChE activity following Ellman’s method [31]. Firstly, cell homogenates were prepared. Briefly, cells were detached from the surface of the 12 well plate with a cell scrapper and centrifuged along with the cell culture medium at 300× *g* for 10 min at room temperature (RT) in a Centric 322B centrifuge (Domel, Železniki, Slovenia). After removing the supernatants, cells were resuspended in 310 µL 0.1% Triton X-100 and put on ice. After that, cells were centrifuged at 10,000× *g* for 10 min at 4 °C in a Sigma 3–30 KS centrifuge (Sigma Aldrich, St. Louis, MO, USA) to separate the membranes and (nano)particles from the sample (supernatant) used for ChE measurements. For ChE assay, 90 µL of the supernatant (100 mM potassium phosphate (K-P) buffer, pH 8 with 0.1% Triton X-100 for blank) was transferred into a 96 well microtiter plate with 90 µL of Ellman’s reagent (5,5′-dithiobis-(2-nitrobenzoic acid) (DTNB)) in 250 mM potassium phosphate buffer (P-P buffer) (100 mM, pH 8.0), pH 7.4) and left for 20 min. After 20 min, the endogenous reaction of cell substrates, usually present in the sample, was completed. After 20 min, 20 µL of 1 mM substrate acetylthiocholine chloride was added to each well, and absorbance values were measured at 420 nm using a spectrophotometer (BioTek, Cytation 3, Bad Friedrichshall, Germany) for 20 cycles (at 1 min intervals, for 20 min). All measurements were performed at RT in triplicates. The experiment was performed in three repetitions. The activity of ChE was expressed as nM/min/mg of proteins, therefore also the concentration of proteins was measured in each sample using the Protein Kit Pierce™ BCA Protein Assay Kit (Thermo Fischer Scientific, Waltham, MA, USA). 

For protein assay, 20 µL of sample (100 mM K-P buffer, pH 8 with 0.1% Triton-X 100 for blank) was transferred into a 96 well microtiter plate with 200 µL of mixture of reagents A:B (50:1) from the kit and incubated at 37 °C for 30 min. After incubation absorbance at 560 nm using a spectrophotometer (BioTek, Cytation 3, Bad Friedrichshall, Germany) was measured in triplicate. Concentration of proteins in mg/mL was calculated using a standard curve made with measuring of the BSA standards (2, 1.5, 1, 0.75, 0.5, 0.25, 0.125 and 0 mg/mL).

### 2.9. Glutathione S-Transferase Activity Assay

The sample was prepared the same way as for the ChE assay. For the GST assay, we followed the Mannervik method [32]. A total of 50 µL of the supernatant (100 mM K-P buffer, pH 8 with 0.1% Triton-X 100 for blank) was transferred into a 96 well microtiter plate with 50 µL of 4 mM 1-Chloro-2,4-dinitrobenzene (CDNB) (Sigma Aldrich, St. Louis, MO, USA), prepared in absolute ethanol and 50 µL of 4 mM L-glutathione reduced (GSH) (Sigma-Aldrich, St. Louis, MO, USA), prepared in K-P buffer, pH 8. Absorbance values were measured at 340 nm using a spectrophotometer Cytation 3 (BioTek, Bad Friedrichshall, Germany) for 20 cycles (at 1 min intervals, for 20 min). The activity of GST was expressed as nM/min/mg of proteins, therefore also the concentration of proteins was measured in each sample using the same protocol as described for the ChE assay. All measurements were performed at RT in triplicates. The experiment was performed in three repetitions.

### 2.10. Detection of Reactive Oxygen Species (ROS), Lipid Droplets (LD) and Apoptosis via Flow Cytometry

We followed the procedures described in [33]. Positive controls cells for LD and apoptosis were treated with 75 µM oleic acid (Cayman Chemical, Ann Arbor, MI, USA) or Staurosporine 10 µM for 24 h, respectively. Cells were then harvested and centrifuged at 300 g, for 10 min at RT. Subsequently, cells were re-suspended in PBS (Sigma-Aldrich) and 5 mM 2′,7′-Dichlorodihydrofluorescein diacetate (CM-H2DCFA) (Thermo Fisher Scientific, St. Louis, MO, USA) (for ROS) or 0.5 µg/mL boron dipyrromethene (BODIPY) 483/503 (for LD) and incubated for 30 min at 37 °C (for ROS) or RT (for LD). Then, 1 drop/0.5 mL of Annexin V-Pacific Blue (Annexin V-Pacific Blue Ready Flow Reagent, Thermo Fisher Scientific, St. Louis, MI, USA) was added to the mixture. After 15 min, the cell fluorescence was measured using the flow cytometer FACS Melody (Becton Dickinson Biosciences, Franklin Lakes, NJ, USA) equipped with violet (405 nm), blue (488 nm) and yellow/green (561 nm) lasers. Cells for ROS positive control were also incubated with 5 mM H_2_O_2_ for 30 min after staining and before measurement. For cytotoxicity, we used the Annexin V-Pacific Blue Ready Flow Reagent (Thermo Fisher Scientific, St. Louis, MO, USA) staining kit to monitor apoptosis using flow cytometry. Samples were stained for cytotoxicity when prepared for ROS and LD detection.

### 2.11. Scanning Electron Microscopy (SEM)

The samples were fixed with OsO_4_ as adapted from [34]. The samples were fixed on 0.05 micron mixed cellulose ester filters (Sterlitech, Auburn, NJ, USA). The samples were incubated in 1% OsO_4_ for 2 h, washed 3 times with distilled water (incubation time 10 min each) then dehydrated in graded series of ethanol (30%, 50%, 70%, 80%, 90% and “absolute”, 10 min each; absolute ethanol was replaced 2 times) and hexamethyldisilazane (mixed with absolute ethanol; 30%, 50% and “absolute”, 10 min each). Then, the samples were left overnight to dry in air. For examination under a JSM-6500F Field Emission Scanning Electron Microscope (JEOL JSM-6500F, Tokyo, Japan) (accelerating voltage 15 kV, working distance 10 mm, beam current 700 pA) the samples were coated (7 nm) with Au/Pd by Gatan 682 Etching and Coating System (PECS) (Pleasanton, CA, USA). 

### 2.12. Statistical Analysis

The data from IL-6, IL-1β, TNF-α, ChE and GST measurements were expressed as arithmetic means ± standard deviations (SD) and were statistically analyzed with one-way analysis of variance (ANOVA), followed by Dunnett’s multiple comparison test. All the statistical analyses were made with Prism 5.03 Software (GraphPad Software, Boston, MA, USA).

## 3. Results

### 3.1. Characterization of Particles

**Figure 1:**
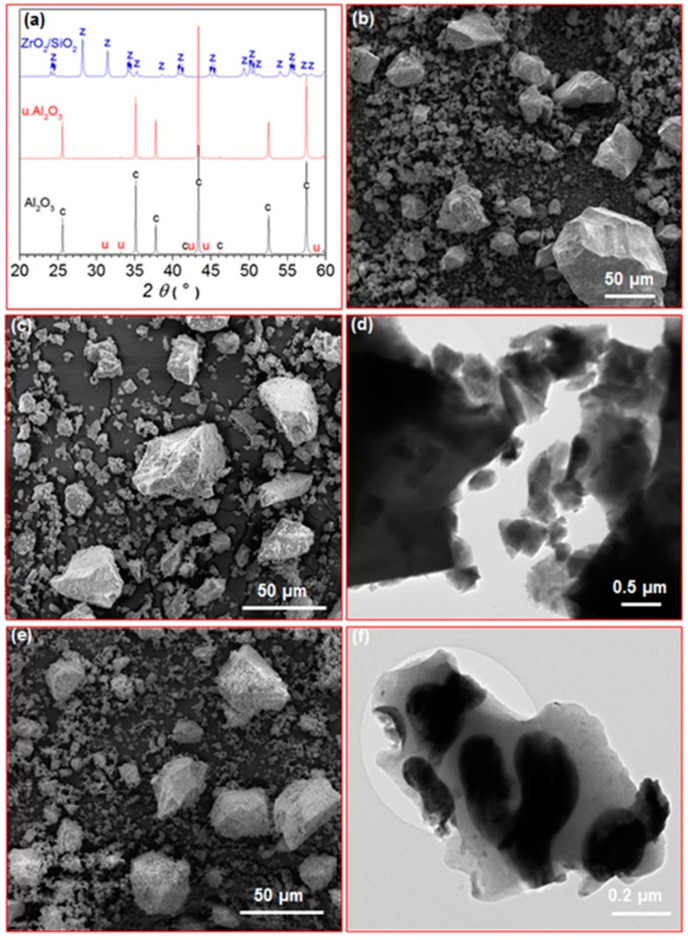
(**a**) XRD patterns of Al_2_O_3_, u. Al_2_O_3_ and ZrO_2_/SiO_2_ abrasives (c: corundum, u: unknown, z: monoclinic zirconia); (**b**,**c**) SEM images of Al_2_O_3_ and u. Al_2_O_3_ samples, respectively; (**d**) TEM image of Al_2_O_3_ sample; (**e**) SEM image of ZrO_2_/SiO_2_ sample; (**f**) TEM image of ZrO_2_/SiO_2_ sample.

XRD for the Al_2_O_3_ and u. Al_2_O_3_ samples appeared very similar. The XRD patterns showed strong, sharp reflections corresponding to corundum and very weak reflections, which could not be identified (Figure 1a). Moreover, the morphology of both Al_2_O_3_ and u. Al_2_O_3_ samples was similar. SEM showed particles of irregular shapes with sizes ranging from several tens of nm to several tens of µm (Figure 1b,c). TEM showed that the smallest nanoparticles from the Al_2_O_3_ and u. Al_2_O_3_ samples were approximately 50 nm in size (Figure 1d). EDXS showed the presence of only Al and O in the unused Al_2_O_3_, whereas in the u. Al_2_O_3_ sample, P, Ca, Na, Cl and Ag were also detected as minor elements. In the ZrO_2_/SiO_2_, monoclinic zirconia was the only crystalline phase detected by XRD (Figure 1a). Moreover, the ZrO_2_/SiO_2_ sample consisted of irregularly shaped particles with a very broad size distribution ranging from less than hundred nm to several tens of µm; however, the particles had much rougher surfaces compared to the Al_2_O_3_ and u. Al_2_O_3_ abrasives (Figure 1e). TEM showed that that the particles of the ZrO_2_/SiO_2_ abrasive were composed of elongated crystalline zirconia particles embedded in the amorphous silica matrix (Figure 1f). EDXS analysis showed only Zr and O at the crystalline areas and Si, O, Al and Zr at the amorphous areas.

Table 1 shows the parameters of the characterization of particle suspensions: zeta potential, pH and average hydrodynamic particle diameter (D_h_) measured in fresh and conditioned cell media. Zeta potential was slightly negative in all samples measured. Two populations of particles corresponding to two peaks of the I(D_h_) curve were detected: a population of small particles with average D_h_ below 10 nm and a population of particles with average D_h_ larger than 1 μm (Table 1).

### 3.2. Morphological Changes of the Treated Cells

**Figure 2:**
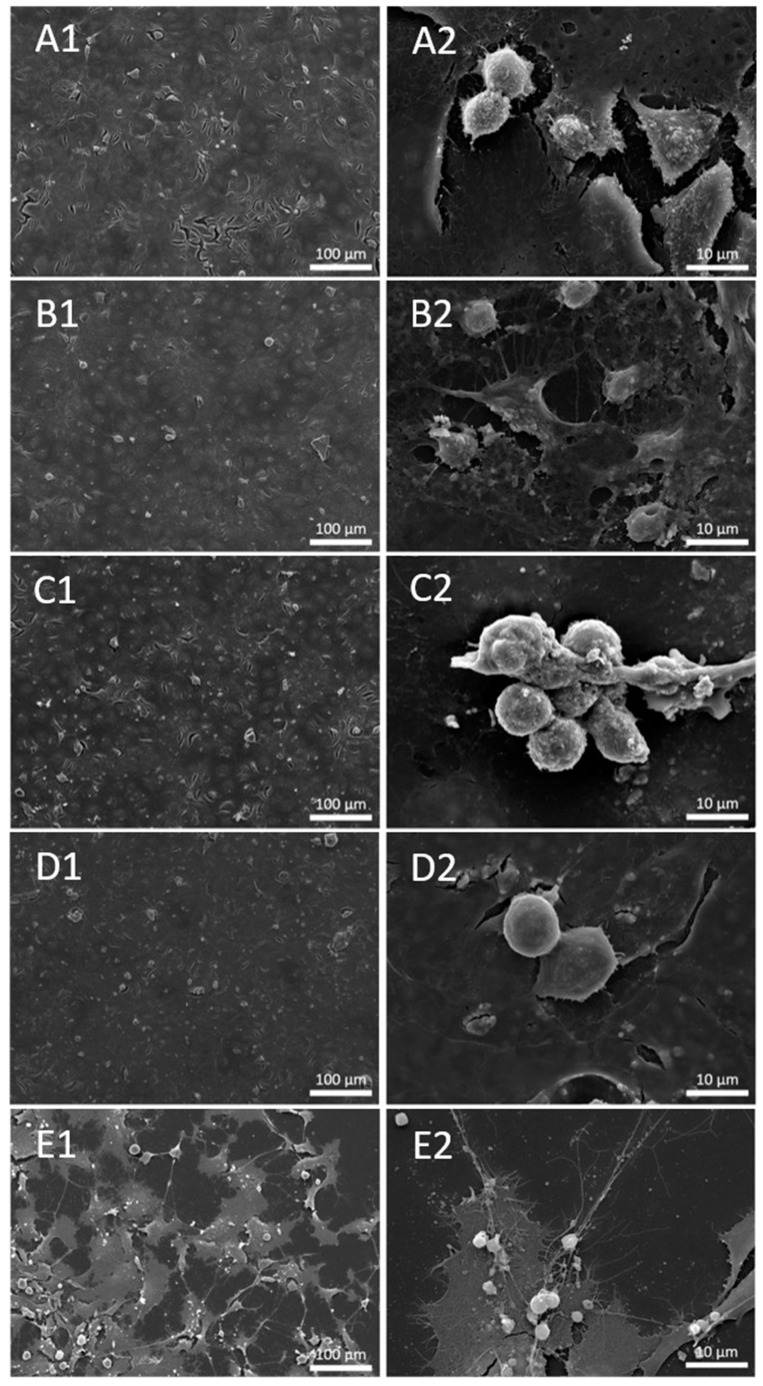
Secondary Electron (SE) image of untreated cells (**A1**,**A2**) and cells treated with 100 μg/mL concentration of u. Al_2_O_3_ (**B1**,**B2**), Al_2_O_3_ (**C1**,**C2**), ZrO_2_/SiO_2_ (**D1**,**D2**) and positive control—Staurosporine (**E1**,**E2**).

SEM analysis revealed that cells treated with the three types of material did not differ in morphology or surface coverage when compared to untreated cells. In contrast, as expected, the positive control treated with the apoptosis inducer staurosporin was strongly affected in both parameters (Figure 2).

### 3.3. Inflammatory Response of HUVEC Cells

**Figure 3:**
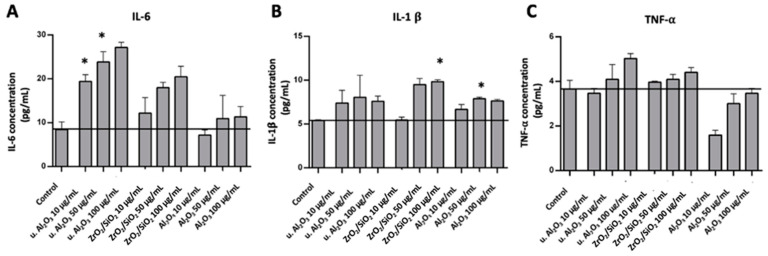
Concentration of IL-6 (**A**), IL-1β (**B**) and TNF-α (**C**) in conditioned media of cells treated with three different types of particles (u. Al_2_O_3_, ZrO_2_/SiO_2_ and Al_2_O_3_). Particles were administered at three concentrations (10 μg/mL, 50 μg/mL and 100 μg/mL). The horizontal line denotes the average concentration of negative control samples (untreated cells). Experiments were performed in duplicate and bars represent the standard deviations. Asterisks denote statistically significant differences with respect to the control.

To assess the inflammatory response of HUVEC, IL-6, IL-1β and TNF-α were measured in conditioned media of HUVEC after 24 h exposure of the cells to three different types of particles at three concentrations (10, 50 and 100 μg/mL). An increase in IL-6 with respect to control was observed in samples treated with u. Al_2_O_3_ and ZrO_2_/SiO_2_ at all three concentrations (Figure 3A). In samples treated with unused Al_2_O_3_ the effect was the least; for the lowest concentration the effect was within the experimental error of the control, while the higher concentrations of Al_2_O_3_ likewise increased the concentration of IL-6 in the conditioned medium (Figure 3A). These results indicate that all types of particles tested induced an increase of IL-6 concentration in the conditioned media. The effect of the particles on the concentration of IL-1β in the conditioned media was less pronounced than that of IL-6; it stayed within the experimental error for u. Al_2_O_3_ particles with concentrations 10 and 50 μg/mL (Figure 3B). However, at higher concentrations of ZrO_2_/SiO_2_ and unused Al_2_O_3_, an increase in the concentration of IL-1β was noted (Figure 3B). TNF-α concentration increased in samples treated with ZrO_2_/SiO_2_ at 50 μg/mL and 100 μg/mL and in samples treated with u. Al_2_O_3_ at all three concentrations (Figure 3C). For both ZrO_2_/SiO_2_ and u. Al_2_O_3_, a concentration-dependent trend was observed. TNF-α concentration was not increased in samples treated with unused Al_2_O_3_ (Figure 3C). 

### 3.4. Oxidative Stress Response of HUVEC Cells

**Figure 4:**
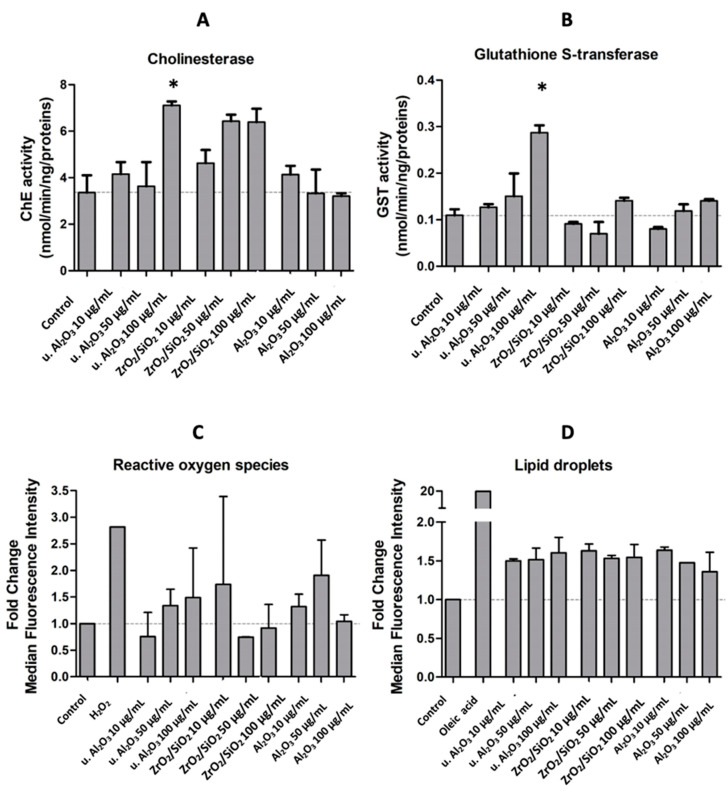
ChE activity (**A**), GST activity (**B**), fold change of median fluorescence intensity of ROS (CM-H_2_DCFA) (**C**) and LD (BODIPY 483/503) (**D**) in comparison to controls of HUVEC cells treated with either of the three different types of particles (u. Al_2_O_3_, ZrO_2_/SiO_2_ and Al_2_O_3_) at three concentrations (10 μg/mL, 50 μg/mL and 100 μg/mL), or the positive controls (5 mM H_2_O_2_ and 75 µM oleic acid). Experiments were performed in triplicate (**A**,**B**) or duplicate (**C**,**D**) and bars represent the standard deviations. Asterisks denote statistically significant differences with respect to the control.

To assess the oxidative stress response of HUVEC, activities of ChE and GST and quantities of ROS and LD were measured after 24 h exposure of HUVEC to three different types of particles at three concentrations (10 μg/mL, 50 μg/mL and 100 μg/mL). ChE and GST activities were expressed as activity in nmol/min/ng/proteins. ROS and LD production were expressed by the fold change of median fluorescence intensities of the respective dyes in comparison to control cells. The average ChE activities of all tested samples, except 50 μg/mL and 100 μg/mL of unused Al_2_O_3_, were higher than the average ChE activity of the control; however, the experimental errors were rather large. The only statistically significant difference with respect to the control was observed with 100 μg/mL of u. Al_2_O_3_ (Figure 4A). The activity of GST was higher in samples treated with u. Al_2_O_3_ in a concentration-dependent way (Figure 4B). A slight increase of GST activity was observed when cells were treated with 100 μg/mL of ZrO_2_/SiO_2_ and with u. Al_2_O_3_ at higher concentrations (Figure 4B). The amount of ROS was increased in all samples except for two (samples treated with u. Al_2_O_3_ at 10 μg/mL and with ZrO_2_/SiO_2_ at 50 μg/mL) (Figure 4C). For u. Al_2_O_3,_ a concentration-dependent trend was observed (Figure 4C). The number of LDs was increased in all treated samples in comparison to untreated samples (Figure 4D). No clear LD concentration-dependent trend was observed (Figure 4D). A clear increase in ROS and LD production was successfully induced in positive control samples after treatment with 5mM H_2_O_2_ and 75 µM oleic acid.

**Figure 5:**
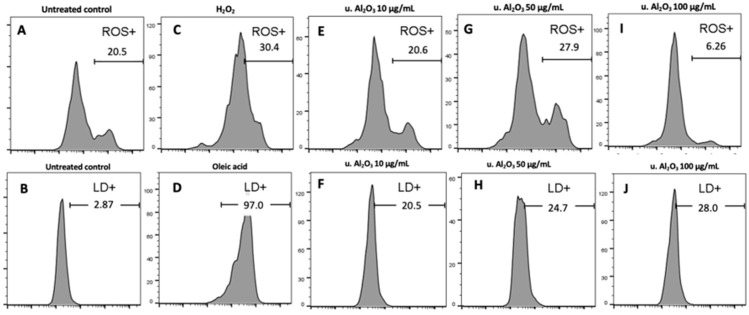
Top: histograms showing ROS production due to treatment with u. Al_2_O_3_. Bottom: histograms showing LD production due to due to treatment with u. Al_2_O_3_. (**A**,**B**) untreated cells, (**C**) positive controls for ROS—cells treated with 5 mM H_2_O_2_, (**D**) positive controls for lipid droplets (LD)—cells treated with 75 μM oleic acid, (**E**) cells treated with 10 μg/mL u. Al_2_O_3_ (ROS), (**F**) cells treated with 10 μg/mL u. Al_2_O_3_ (LD), (**G**) cells treated with 50 μg/mL u. Al_2_O_3_ (ROS), (**H**) cells treated with 50 μg/mL u. Al_2_O_3_ (LD), (**I**) cells treated with 100 μg/mL u. Al_2_O_3_ (ROS), (**J**) cells treated with 100 μg/mL u. Al_2_O_3_ (LD). For ROS detection, cells were stained with CM-H_2_DCFDA; for LD detection, cells were stained with BODIPY 493/503. Gates indicate the % of ROS and LD-positive cells. Abscissa gives the fluorescence intensity of the signal release by the fluorophores, and ordinate gives the number of events (cells) detected by the flow cytometer (FCM) for a given intensity.

Since u. Al_2_O_3_ particles were shown to be the most bioactive in terms of ChE, GST and IL-6 production, we chose them to study the ROS production and apoptosis in cells. The histograms (Figure 5) show the fluorescence of cells stained with CM-H_2_DCFA (ROS) and BODIPY 483/503 (LD). Gates (horizontal bars) indicate the % of cells treated with u. Al_2_O_3_ that were positive for ROS or LD in comparison to the untreated control. Untreated cells were 20% positive for ROS and slightly positive for LD (Figure 5A,B). For the ROS-positive control, samples were treated with 5 mM H_2_O_2_ resulting in 30.4% of positive cells (Figure 5C). For the LD-positive control, 75 μM oleic acid was used, resulting in 97.0% of positive cells (Figure 5D). A total of 27% of cells treated with u. Al_2_O_3_ were ROS-positive when particles were used at a concentration of 50 μg/mL. In contrast, at 10 μg/mL concentration of u. Al_2_O_3_, fewer treated cells than control cells were ROS positive. All particle-treated samples were positive for LD production in comparison to negative control samples (~20% vs. 2%), although in a concentration-independent manner (Figure 5B,F,H,J).

### 3.5. Cytotoxicity

**Figure 6:**
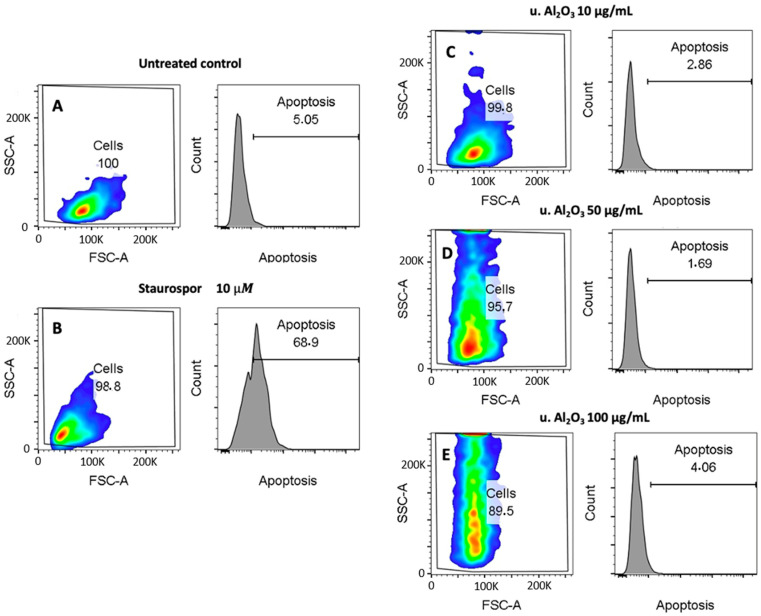
Cytotoxicity effect of u. Al_2_O_3_ particles on HUVEC. (**A**) untreated cells, (**B**) positive control for apoptosis (cells treated with Staurosporine), (**C**) HUVEC treated with 10 μg/mL of u. Al_2_O_3_, (**D**) HUVEC treated with 50 μg/mL of u. Al_2_O_3_, (**E**) HUVEC treated with 100 μg/mL of u. Al_2_O_3_. SSC-A—side scatter measurements; information on internal complexity (granularity), FSC-A—forward scatter; information on cell size. The numbers in boxes represent percent of cells in the respective gates (left) and percent of apoptotic cells (right).

Apoptosis may play an important role in the regulation of inflammation or be the result of inflammation in cells. Figure 6 shows both FSC/SSC dot plots and Annexin V fluorescence histograms (marker for apoptosis) of untreated cells (negative control), Staurosporine-treated cells (positive control) and u. Al_2_O_3_-treated cells. By comparing untreated controls (Panel A) with the positive control (Panel B), it can be seen that Staurosporine effectively expanded the apoptotic population (69% vs. 5%) during the 24 h treatment. In contrast, no significant differences are visible between the negative control (Panel A) and particle-treated samples (Panels C−E), indicating that no apoptosis was induced upon 24 h of incubation with u. Al_2_O_3_. In the dot plots shown in Figure 6, a dose-dependent increase of the SSC signal can be observed. Increase in cell granularity could be a consequence of particle endocytosis or LD/intracellular vesicle formation. This would be in line with the data presented in Figure 4 and Figure 5, which show an increase in LD formation. 

## 4. Discussion

We have treated the HUVEC with three types of particles (Al_2_O_3_, u. Al_2_O_3_ (retrieved from removed OI) and ZrO_2_/SiO_2_). We observed no morphological changes of cells treated with Al_2_O_3_, ZrO_2_/SiO_2_ and u. Al_2_O_3_ compared to untreated control cells (Figure 2). These findings were further supported by the data obtained via flow cytometry showing that no significant changes in cell viability were visible in samples treated in comparison to the negative control samples. We observed an increase in inflammation parameter IL-6 after treatment with all types of particles and there was a concentration-dependent trend after treatment with u. Al_2_O_3_ and ZrO_2_/SiO_2_. We observed an increase in the inflammation parameter IL-1β with a concentration-dependent trend after treatment with ZrO_2_/SiO_2_ (Figure 3) and after treatment of cells with 50 µg/mL Al_2_O_3_. We detected an increase of the oxidative stress parameters: ChE and GST activities were significantly higher in samples treated with 100 µg/mL u. Al_2_O_3_ (Figure 4). We found a trend of increasing quantities of LD in samples treated with all concentrations of all types of particles (Figure 4 and Figure 5), which could be a consequence of oxidative stress. LD can play a protective role against ROS [24]. ROS, which are commonly produced during cell metabolism, were not significantly increased in monitored cells (Figure 4 and Figure 5). Moreover, we observed no cytotoxicity effects. OIs are implanted for years, indicating a release of wear debris in the surrounding tissue and in blood circulation. In contrast, the time of treatment of cells in our study was 24 h, which could show an acute response of the cells to particle exposure only. For times shorter than 24 h, the expected effect would be smaller. For longer maintenance of cells the medium should have been changed, which would remove the particles and disturb the effect that we wished to observe. Nevertheless, our results show that particles are not inert as regards the cellular response. This was the aim of the present work, however, further study (including the time dependence) of the effect of the particles is indicated. To decisively point to inflammation, oxidative stress and increased cell vesiculation, experiments should include time dependence of the effects in vitro in different types of cells, and observation of short- and long-term effects in vivo. Another limitation of the study is that the size of the particles has not been taken into account. 

In the literature, eventual implant loosening due to aseptic osteolysis has been attributed to local inflammatory responses to wear and corrosion products that are produced by articulating implant interfaces [20,21]. The response to implant debris is dominated by local immune activation, e.g., macrophages [11,12,16,35]. Generally, to produce an in vitro inflammatory response, particles need to be less than 10 μm in size, i.e., prone to being phagocytosed. Immune reactivity has been shown to depend on the number of particles produced or the dose (i.e., the concentration of phagocytosed particles per tissue volume, which can be characterized by knowing the size distribution and the number of debris) [12]. Evidence involving cells, model particles and pathogenic microbes indicates that particle size, shape, rigidity and surface roughness are important parameters for cellular uptake and subsequent immune responses [36]. In interaction of particles with the membrane, the (mis)match of the membrane curvature and the intrinsic curvature of the particle are key factors which dictate how likely it is that the particle would be taken up by the cell [37]. Elongated particles (fibers) are generally more pro-inflammatory than round particles, and there is a growing consensus that metallic particles are more proinflammatory than polymers in vivo [12]. It was found that titanium dioxide nanoparticles (TiO_2_ NPs) induced oxidative stress, reduced osteogenesis and impaired the antioxidant defense system [38]. Jamieson et al. (2021) reported that ceramic oxide nanopowders in vitro were phagocytosed by THP-1 macrophages, which resulted in a cell inflammatory response [39]. A significant increase in IL-1β secretion of the cells following ceramic treatment was observed [39]. Bertrand et al. (2018) reported fibrotic changes and the presence of ceramic wear particles in periprosthetic tissue around ceramic-coated OI [40]. They found a correlation between increased tissue fibrosis and implantation time and therefore assumed that induction of fibrosis was connected to the release of debris from OI. This indicated that ceramic OI produces wear that could be biologically active [40]. They suggested that the reason for tissue fibrosis might have been the long-term inflammatory response of peripheral blood mononuclear cells and the inflammatory response of fibroblasts to ceramic OI. Moreover, they observed in vitro effects of ceramics on peripheral blood mononuclear cells from healthy donors during two days of incubation [40], short-term inflammatory effects and inflammatory response by an increased IL-1β and IL-6 concentrations. This agrees with our results (Figure 3). In contrast, Bylski et al. [41] found no increase in TNF-α concentration in THP-1 monocytic cells treated with aluminum oxide. 

Toxicity of the released particles to cells can be on a chemical basis (due to the released soluble ions or molecules) or on a mechanical basis (due to mechanical impact of the insoluble particles). It was found that cytotoxicity of ceramic particles to macrophages was lower than that corresponding to metal ions and that it did not depend on their chemical species [42]. Moreover, while the differences in size of the particles did not affect their mechanical toxicity, the shape of the particles mattered: the dendritic particles had a higher cytotoxicity than spindle and globular particles [42]. Ceramics might induce different cytotoxic effects in different cell types and that some cell types are more sensitive when encountering ceramic particles. Cytotoxicity of insoluble wear debris (e.g., Al_2_O_3_ particles) was reported to be lower than the wear debris of soluble metal ions [39], which were also shown to cause oxidative stress [43,44]. Moreover, Yamamoto et al. (2004) reported that particle shape influenced particle cytotoxicity [42]. The most toxic were dendritic-shaped particles, followed by spindle- and globular-shaped particles [42]. Cytotoxicity of cells exposed to Al_2_O_3_ nanoparticles was previously discussed by Radziun et al. [45], who reported that Al_2_O_3_ nanoparticles could penetrate the membranes of L929 mouse fibroblasts and BJ human fibroblasts, yet decrease in cellular viability was not detected. Similar results were reported by Jamieson et al. [39] for THP-1 human macrophages. It was concluded that, even though cell lines could phagocytose Al_2_O_3_ nanoparticles, no significant cytotoxic effects were observed [39]. Moreover, our results did not suggest cytotoxic effects of Al_2_O_3_ nanoparticles on cell line HUVEC after 24 h exposure (Figure 6). In contrast, Catelas et al. (1999) reported a higher rate of apoptosis of the J774 macrophages for larger Al_2_O_3_ particles (4.5 μm in diameter), while smaller particles (0.6, 1.3 and 2.4 μm in diameter) had less effect [46]. Olivier et al. (2003) tested the cytotoxicity of J774.2 macrophages and L929 fibroblasts after treatment with Al_2_O_3_ particles [47]. Particles were more cytotoxic for macrophages than for fibroblasts, having an impact on apoptosis and necrosis of the cells [47]. Significant decrease in the viability of 3T3-E1 mouse osteoblast-like cells after treatment with zirconia nanoparticles was reported by Ye et al. (2018) [48]. Xie et al. (2019) observed that Al_2_O_3_ was unable to achieve good chemical bonding with tissues [49]. To overcome this problem, they prepared a new material, SiAlON–Al_2_O_3_ ceramics, with a porosity and compression strength that is more suitable for proliferation and survival of cells [49]. 

With SEM (Figure 2) we wanted to observe budding of the plasma membrane of HUVEC, yet there was no visible plasma membrane budding of treated or untreated cells. Budding precedes the formation of small cellular particles (SCPs) that become free to move in the surrounding medium and are, in principle, able to reach distant cells if they are transported by body fluids. Although the mechanisms of SCP uptake by cells are not completely understood, it has been reported that SCPs may affect the phenotype and function of the recipient cells [50,51]. Methods have been developed to harvest SCPs from the cell culture media [52,53,54], e.g., differential centrifugation, size exclusion chromatography, immune-capture-based methods and microfluidic device-based methods. SCPs are heterogeneous in shape and composition; furthermore, if they are membrane enclosed, their identity is not fixed and they are subject to transformations of shape, size and composition during the processing of samples [55,56]. Although much effort has been invested in the elaboration of SCP harvesting from different media and body fluids, there are still fundamental issues associated with these procedures [57]. We have performed the isolation of SCPs from the media in which the cells had grown by differential ultracentrifugation. We have used a protocol that is common for harvesting small extracellular vesicles [58]. We have assessed samples by interference light microscopy and visualized them by cryogenic electron microscopy [59]. The signal was under the detection limit of the interference light microscopy, and we observed no membrane-enclosed particles by cryogenic electron microscopy (not shown). SEM images (Figure 2), confirm our inability to detect free SCPs with other used methods. As SCPs have recently been gaining interest for their biological roles, budding and vesiculation of the cells induced by particles should be further studied in the future. 

## 5. Conclusions

We observed changes in morphology and in inflammation markers (IL-6 and IL-1β concentration), and oxidative stress parameters (ChE and GST activity and LD quantities) in HUVEC after 24 h of treatment with some types of particles. Within the range of the studied parameters, we found no evidence of their cytotoxic effects. We have not observed increased membrane budding in the treated cells and we found no small cellular particles in the isolates from the media. Further studies on the effect of particles are indicated by observing longer treatment and by exploring the non-local effects emerging from small cellular particles possibly shedding off differently from the treated cells.

## Figures and Tables

**Table 1:** Zeta potential and DLS assessment of particle solutions.

Sample	u. Al_2_O_3_		Al_2_O_3_		ZrO_2_/SiO_2_	
Conditioned (C)/Fresh(F) medium	C	F	C	F	C	F
pH	7.87	7.87	7.87	8.19	7.87	7.87
Zeta potential [mV]	−10.3	−10.4	−10.4	−12.1	−10.7	−10.7
D_h_ [nm] (1st peak)	1085	2055	2055	1538	1316	1316
D_h_ [nm] (2nd peak)	6	14	14	6	6	6

## Data Availability

Raw data are presented as Supplementary Materials.

## Supplementary Materials

The following supporting information can be downloaded at: https://www.mdpi.com/article/10.3390/ma16093287/s1.
