# Supplementary material for: Inflammatory, Oxidative Stress and Small Cellular Particle Response in HUVEC Induced by Debris from Endoprosthesis Processing
Source: Materials (Basel). 2023 Apr 22;16(9):3287. doi: 10.3390/ma16093287 (PMC10179554; doi:10.3390/ma16093287)
Supplement: Supplementary file 1 [file materials-16-03287-s001.zip › materials-2336980-supplementary.pdf]

Raw data

Legend

A: absorbance (arbitrary unit a.u.)

c: concentration (pg/mL)

IL-6

Sample	A1	A2	A	STD A
Control	0.1190	0.1220	0.1205	0.0021
U. Al2O3 10 µg/mL	0.1240	0.1350	0.1295	0.0078
U. Al2O3 50 µg/mL	0.1370	0.1290	0.1330	0.0057
U. Al2O3 100 µg/mL	0.1420	0.1440	0.1430	0.0014
ZrO2/SiO2 10 µg/mL	0.1200	0.1180	0.1190	0.0014
ZrO2/SiO2 50 µg/mL	0.1190	0.1280	0.1235	0.0064
ZrO2/SiO2 100 µg/mL	0.1220	0.1260	0.1240	0.0028
Al2O3 10 µg/mL	0.1220	0.1280	0.1250	0.0042
Al2O3 50 µg/mL	0.1190	0.1330	0.1260	0.0099
Al2O3 100 µg/mL	0.1330	0.1370	0.1350	0.0028

Calibration

c IL-6	A1	A2	A	STD A
0	0.112	0.112	0.1120	0.0000
1.37	0.116	0.115	0.1155	0.0007
4.12	0.116	0.114	0.1150	0.0014
12.35	0.125	0.117	0.1210	0.0057
37.04	0.154	0.147	0.1505	0.0049
111.1	0.265	0.256	0.2605	0.0064
333.3	0.542	0.455	0.4985	0.0615
1000	1.293	1.354	1.3235	0.0431
A= 0.0012 a.u. mL/pg c + 0.1104 a.u.				

IL-1 beta

Sample	A1	A2	A	STD A
Control	0.1560	0.1520	0.1540	0.0028
U. Al2O3 10 µg/mL	0.1880	0.1630	0.1755	0.0177
U. Al2O3 50 µg/mL	0.1620	0.2050	0.1835	0.0304
U. Al2O3 100 µg/mL	0.1730	0.1830	0.1780	0.0071
ZrO2/SiO2 10 µg/mL	0.1590	0.1370	0.1480	0.0156
ZrO2/SiO2 50 µg/mL	0.1810	0.1700	0.1755	0.0078
ZrO2/SiO2 100 µg/mL	0.2680	0.2640	0.2660	0.0028
Al2O3 10 µg/mL	0.1490	0.1550	0.1520	0.0042
Al2O3 50 µg/mL	0.1970	0.2050	0.2010	0.0057
Al2O3 100 µg/mL	0.2040	0.2070	0.2055	0.0021

Calibration

c IL-1 b	A1	A2	A	STD A
0	0.074	0.111	0.0925	0.0262
0.48	0.132	0.151	0.1415	0.0134
1.02	0.179	0.208	0.1935	0.0205
2.56	0.239	0.334	0.2865	0.0672
6.4	0.425	0.521	0.4730	0.0679
16	0.773	0.832	0.8025	0.0417
40	1.33	1.287	1.3085	0.0304
100	1.763	1.738	1.7505	0.0177
A = 0.0121 a.u. mL/pg c + 0.0861 a.u.				

TNF alpha

Sample	A1	A2	A	STD A
Control	0.0610	0.0560	0.0585	0.0035
U. Al2O3 10 µg/mL	0.0600	0.0590	0.0595	0.0007
U. Al2O3 50 µg/mL	0.0630	0.0600	0.0615	0.0021
U. Al2O3 100 µg/mL	0.0640	0.0650	0.0645	0.0007
ZrO2/SiO2 10 µg/mL	0.0540	0.0530	0.0535	0.0007
ZrO2/SiO2 50 µg/mL	0.0590	0.0570	0.0580	0.0014
ZrO2/SiO2 100 µg/mL	0.0590	0.0600	0.0595	0.0007
Al2O3 10 µg/mL	0.0610	0.0580	0.0595	0.0021
Al2O3 50 µg/mL	0.0610	0.0620	0.0615	0.0007
Al2O3 100 µg/mL	0.0620	0.0630	0.0625	0.0007

Calibration

c TNF	A1	A2	A	STD A
0.00	0.0110	0.0320	0.0215	0.0148
15.60	0.0920	0.0850	0.0885	0.0049
31.20	0.1660	0.1700	0.1680	0.0028
61.50	0.3200	0.3100	0.3150	0.0071
125.00	0.4800	0.4820	0.4810	0.0014
250.00	0.7300	0.7000	0.7150	0.0212
500.00	1.7400	1.6600	1.7000	0.0566
A = 0.0032 a.u. mL/pg c + 0.0484 a.u.				

Legend

A: Absorbance (arbitrary unit a.u.)

K = dA/dt (a.u./min)

c: concentration (mg/mL)

ChE

Sample	K1	K2	K3	K	STD K
Control	0.0412	0.0480	0.0440	0.0444	0.0034
U. Al2O3 10 µg/mL	0.0285	0.0279	0.0310	0.0291	0.0016
U. Al2O3 50 µg/mL	0.0515	0.0669	0.0626	0.0603	0.0080
U. Al2O3 100 µg/mL	0.0566	0.0561	0.0557	0.0561	0.0004
ZrO2/SiO2 10 µg/mL	0.0341	0.0333	0.0329	0.0335	0.0006
ZrO2/SiO2 50 µg/mL	0.0238	0.0291	0.0284	0.0271	0.0029
ZrO2/SiO2 100 µg/mL	0.0366	0.0381	0.0373	0.0373	0.0007
Al2O3 10 µg/mL	0.0417	0.0414	0.0496	0.0442	0.0047
Al2O3 50 µg/mL	0.0531	0.0617	0.0577	0.0575	0.0043
Al2O3 100 µg/mL	0.0976	0.0967	0.0953	0.0965	0.0012

Sample	A	c
Control	0.30545	0.3226
U. Al2O3 10	0.2039	0.1960
U. Al2O3 50	0.34235	0.3685
U. Al2O3 100	0.2047	0.1970
ZrO2/SiO2 10	0.0645	0.0223
ZrO2/SiO2 50	0.12655	0.0996
ZrO2/SiO2 100	0.2731	0.2822
Al2O3 10	0.26125	0.2675
Al2O3 50	0.2366	0.2368
Al2O3 100	0.35445	0.3836

GST

Sample label	K1	K2	K3	K	STD K
Control	0.01	0.0055	0.0053	0.0054	0.0001
U. Al2O3 10 µg/mL	0.01	0.0053	0.0054	0.0054	0.0001
U. Al2O3 50 µg/mL	0.01	0.0069	0.0068	0.0071	0.0005
U. Al2O3 100 µg/mL	0.01	0.0090	0.0087	0.0088	0.0002
ZrO2/SiO2 10 µg/mL	0.00	0.0043	0.0045	0.0043	0.0002
ZrO2/SiO2 50 µg/mL	0.01	0.0061	0.0066	0.0062	0.0003
ZrO2/SiO2 100 µg/mL	0.01	0.0053	0.0053	0.0053	0.0000
Al2O3 10 µg/mL	0.01	0.0051	0.0049	0.0050	0.0001
Al2O3 50 µg/mL	0.00	0.0036	0.0042	0.0039	0.0003
Al2O3 100 µg/mL	0.01	0.0056	0.0057	0.0058	0.0002

Calibration (bovine serum albumin (BSA))

c BSA	A1	A2	A	A - A(0)
0	0.096	0.103	0.0995	0
0.0625	0.173	0.175	0.174	0.072
0.125	0.241	0.248	0.2445	0.145
0.25	0.371	0.368	0.3695	0.265
0.5	0.612	0.602	0.607	0.499
1.00	1.00	0.989	0.9955	0.886
2.00	1.73	1.722	1.728	1.619
A = 0.8025 a.u. mL/mg c + 0.0466 a.u.				

Legend

MFI: median fluorescence intensity (/µL)

ROS: reactive oxygen species

LD: lipid droplets

	ROS		LD	
	MFI1	MFI2	MFI1	MFI2
Control	486	618	4533	164
ROS+	1742			
LD+			3204	
u. Al2O3 10 µl/mL	214	665	6723	249
u. Al2O3 50 µl/mL	756	692	6405	266
u. Al2O3 100 µl/mL	1044	512	6635	286
ZrO2/SiO2 10 µl/mL	1412	350	7673	257
ZrO2/SiO2 50 µl/mL	364	457	7073	247
ZrO2/SiO2 100 µl/mL	598	373	6476	273
Al2O3 10 µl/mL	722	714	7556	264
Al2O3 50 µl/mL	1155	891	6694	242
Al2O3 100 µl/mL	549	589	6965	194
